# Assessment of multimodal CEST, perfusion and diffusion MRI for predicting clinical outcome of patients with diffuse glioma following surgery at baseline before radiotherapy

**DOI:** 10.1186/s40644-026-01022-y

**Published:** 2026-03-28

**Authors:** Nikolaus von Knebel Doeberitz, Petr Menshchikov, Florian Kroh, Laila König, Svenja Graß, Cora Bauspieß, Philip S. Boyd, Sebastian Regnery, Thomas Zeyen, Stephen Schaumann, Ralf Floca, Daniel Hasson, Moritz Scherer, Martin Bendszus, Wolfgang Wick, Jürgen Debus, Peter Bachert, Mark E. Ladd, Heinz-Peter Schlemmer, Andreas Korzowski, Daniel Paech

**Affiliations:** 1 Division of Radiology, German Cancer Research Center (DKFZ), Heidelberg, Germany; 2 Division of Medical Physics in Radiology, German Cancer Research Center (DKFZ), Heidelberg, Germany; 3 Department of Radiology, Brigham Women’s Hospital, Harvard Medical School, 75 Francis Street, Boston, MA 02115 USA; 4 Department of Radiation Oncology, Heidelberg University Hospital, Heidelberg, Germany; 5 Faculty of Medicine, University of Heidelberg, Heidelberg, Germany; 6 Department of Neurooncology, Center for Neurology and Center for integrated Oncology (CIO) ABCD, University Hospital Bonn, Bonn, Germany; 7 Department of Medical Image Computing, German Cancer Research Center (DKFZ), Heidelberg, Germany; 8 Department of Radiology, Clínica Alemana-Universidad del Desarrollo, Santiago, Chile; 9 Department of Neurosurgery, Heidelberg University Hospital, Heidelberg, Germany; 10 Department of Neuroradiology, Heidelberg University Hospital, Heidelberg, Germany; 11 Department of Neurology, Heidelberg University Hospital, Heidelberg, Germany; 12 Clinical Cooperation Unit Radiation Oncology, German Cancer Research Center (DKFZ), Heidelberg, Germany; 13 Department of Physics and Astronomy, Heidelberg University Hospital, Heidelberg, Germany

**Keywords:** Chemical exchange saturation transfer, Amide proton transfer, Relayed nuclear Overhauser effect, Semi-solid magnetization transfer, APTw, Lorentzian fit, Relaxation compensation, Diffuse glioma, Radiotherapy, Outcome prediction

## Abstract

**Background:**

To assess the predictive value of different chemical exchange saturation transfer (CEST) contrasts, i.e. of the amide proton transfer (APT), relayed nuclear Overhauser effect (rNOE), and semi-solid magnetization transfer (ssMT), as well as of clinical routine perfusion- and diffusion-weighted MRI, in terms of treatment outcome in patients with glioma following surgery at baseline before radiotherapy at 3 T.

**Materials and methods:**

From September 2018 to December 2022, 78 study participants (median age 62 years, 27/78 female) prospectively underwent CEST, diffusion, and perfusion imaging. CEST contrasts were reconstructed for the APT-weighted magnetization transfer ratio asymmetry (APTw_asym_), relaxation-compensated CEST metrics (MTR_Rex_APT, MTR_Rex_NOE, MTR_Rex_MT), and MT_const_. Contrast-enhancing and whole tumor volumes were segmented on T2w-FLAIR and T1w images. Associations of mean contrast values with therapy response were tested using ROC analyses, while relationships with progression-free survival (PFS, median 6.04 months) and overall survival (OS, median 11.58 months), as well as added benefit compared to nCBV and ADC maps, were assessed using dichotomized Cox regression models.

**Results:**

MTR_Rex_APT, MTR_Rex_NOE, and MTR_Rex_MT were associated with therapy response (AUC = 0.82, 0.81, 0.68; all *p* ≤ 0.03), PFS (HR = 2.92, 0.37, 3.40; all *p* ≤ 0.02), and OS (HR = 2.76, 0.63, 8.09; all *p* ≤ 0.05). MT_const_ was correlated with OS (HR = 5.52, *p* < 0.01), while APTw_asym_ was linked to therapy response (AUC = 0.71, *p* = 0.02). MTR_Rex_MT (χ² = 13.71, *p* < 0.01) and MT_const_ (χ² = 5.62, *p* = 0.018) provided each additional value to nCBV for OS prediction.

**Conclusion:**

Relaxation-compensated CEST imaging of the APT, rNOE, and ssMT, as well as conventional APTw_asym_ showed ability to predict treatment outcome, whilst ssMT-weighted imaging provided added benefit for OS prediction in patients with diffuse glioma following surgery at baseline before radiotherapy at 3 T.

**Supplementary Information:**

The online version contains supplementary material available at 10.1186/s40644-026-01022-y.

## Background

Diffuse gliomas are with an incidence of 4–5/100.000 the most common group of malignant primary brain tumors in adults [1]. The World Health Organization (WHO) uses histopathologic features and pathogenetic characteristics (e.g. oncogenic IDH-mutations) for tumor classification [2, 3]. The standard of care for diffuse gliomas encompasses maximum safe resection followed by radio- and chemotherapy. Unfortunately, clinical outcomes remain fatal despite significant advances in the understanding of glioma pathology and ongoing optimizations of treatment protocols [2, 4]. Therefore, accurate patient stratification and prognostication informed not only by pathology but also complemented by diagnostic imaging is critical to guide patient management effectively.

Contrast enhancement on MRI, increases in relative cerebral blood volume (rCBV) on perfusion-weighted imaging, and lower apparent diffusion coefficient (ADC) values on diffusion imaging have been associated with poorer outcomes of patients with glioma [5]. These imaging biomarkers infer the degree of tumor malignancy and aggressiveness indirectly through the measurement of vascular permeability, tissue vascularization, and cellular density among other features [5]. The introduction of positron emission tomography (PET) imaging using radioactively labelled amino acid tracers has further improved the stratification of patients with glioma according to tumor malignancy, therapy response and survival [6]. However, PET-MRI is costly, infrastructure-intensive, and therefore limited to specialized centers [7]. Chemical exchange saturation transfer (CEST) MRI represents another molecular imaging technique that promises added benefit to the above-mentioned imaging biomarkers without the need for additional infrastructure or the application of exogenous contrast agents or tracers [8–10]. CEST imaging of the amide proton transfer (APT) applies selective off-resonance radiofrequency pulses to saturate protons bound in amide bonds of solute peptides, small proteins and small metabolites. Subsequent chemical exchange results in a saturation transfer to bulk water, which can be visualized via conventional MR readout sequences [8–10]. Other related magnetization transfer-based imaging techniques that are used to visualize the density of aliphatic protons bound in myelin of glial tissues and subcellular macromolecules are the relayed Nuclear Overhauser Effect (rNOE) and semi-solid magnetization transfer (ssMT) respectively (also referred to as CEST imaging in the following) [8–10]. Several groups have demonstrated the potential of APT-, rNOE- and ssMT-imaging to predict therapy response and survival in patients with newly diagnosed glioma, and early after completion of radiotherapy (reviewed in [11]). Others have demonstrated the potential of APT-imaging to differentiate tumor progression, pseudoprogression and radiation necrosis after radiotherapy (reviewed in [11]). However, the sensitivity and specificity of different CEST contrasts are - amongst other parameters - strongly dependent on the metrics used for contrast reconstruction from the *Z*-spectrum and the applied magnetic field strengths [8, 12]. Therefore, the purpose of this study was to assess the prognostic value of CEST imaging of the APT, rNOE and ssMT according to Zhou et al. (APTw_asym_) [13], Goerke et al. (MTR_Rex_APT, MTR_Rex_NOE, MTR_Rex_MT) [14] and Mehrabian et al. (MT_const_) [15] to predict therapy response, progression-free survival and overall survival following surgery at baseline before radiotherapy at a clinical field strength of 3 T. Furthermore, the added benefit of CEST contrasts beyond imaging of the rCBV and ADC for PFS and OS prediction was assessed to explore the synergistic potential of CEST contrasts with clinically established predictive imaging markers.

## Methods

### Study design

From September 2018 to December 2022, 94 study participants undergoing treatment at the Department of Radiation Oncology of the University Hospital Heidelberg were enrolled. CEST MRI was performed following surgery at baseline imaging before radiotherapy. Inclusion criteria encompassed pathologically proven diagnosis of diffuse glioma, age ≥ 18 years, Karnofsky Performance Score (KPS) of ≥ 50 and the capacity to consent. Sixteen participants were excluded from the analysis due to heavy motion artifacts (*n* = 6) and incomplete (*n* = 10) data sets. Nineteen participants included in this analysis were also involved in three previously published evaluations of the study cohort, investigating the clinical value of CEST imaging for early therapy response assessment and outcome prediction [16–18]. At the end of data acquisition (May 3rd, 2025), 29/78 evaluable participants were still on study. Three participants were lost to follow-up. 65/78 participants received treatment for first diagnosis of glioma and 13/78 for relapsing disease. Median age at diagnosis was 62.0 years (95%-CI: 58.5; 64.0), and 27/78 evaluable participants were female. See Table S1 for a coherent review of patient characteristics.

Therapy response after completion of radiotherapy and progression-free survival (PFS) were assessed based on longitudinal clinical and MRI data according to the 2023 update of the response assessment in neuro-oncology criteria (RANO 2.0) independently by two board-certified radiologists with eight (NK) and five (DH) years of experience in reading neuro-oncology MRI scans [19]. Follow-up MR imaging was done four to six weeks after completion of radiotherapy and every three months thereafter. PFS was defined as the interval between commencement of radiotherapy and disease progression. Median follow-up was 23.2 months (min.: 0.6; max.: 30.4). Follow-up MRI data was available for 73 study participants. Discordances between the two readers occurred in 26 cases and were resolved in consensus with a third reader (DP) with 12 years of experience. All assessments were additionally reviewed against multidisciplinary tumor board decisions to account for antiangiogenic therapy, treatment modifications and clinical changes. Overall survival (OS) was determined via public registry data (March 3, 2025) and available for 75 of 78 study participants.

### Histopathology

Tissue diagnosis from biopsy or resection was available for all analyzed patients. IDH, ATRX, LOH1p19q, and MGMT status were determined. Tumors were classified according to the WHO 2016 criteria for primary CNS neoplasms [20], due to the enrollment interval from 2018 to 2022. Reclassification according to the 2021 update of the WHO criteria was waived [3], given that the association of CEST contrast with pathology was not investigated.

### Magnetic resonance imaging (MRI)

Imaging was performed on a 3 T MAGNETOM Prisma scanner (Siemens Healthineers) using a 64-channel head/neck coil.

### CEST MRI

All CEST related measurements were performed using a 3D spiral-centric-reordered gradient-echo sequence (snapshot CEST) with TE = 2.75 ms, TR = 5.5 ms, and 1.7 × 1.7 × 3 mm³ resolution for image readout [21], described in detail in [14]. 16 slices with a thickness of 3 mm and an inter-slice distance factor of 0.2 were acquired, resulting in a cranial-caudal spatial coverage of approx. 57.6 mm.

B_0_ and B_1_ maps were obtained using a WASABI presaturation (3:41 min) [22].

*Low power CEST*: Presaturation employed 148 Gaussian–shaped RF pulses (B_1_ = 0.6 µT and 0.9 µT, t_sat_ = 3.7 s). Fifty-seven unevenly distributed offsets (± 250 ppm) and two M_0_ references (–300 ppm) were acquired (7:34 min/scan) [14]. Data were rigidly registered, B_0_-corrected, and PCA-denoised [23]. CEST data were processed with in–house Matlab^®^ scripts (Mathworks, v2024b).

Two fitting approaches were applied:

1. Four-pool Lorentzian fit (APT, rNOE, ssMT, water) with spillover/MT correction, yielding MTR_Rex_APT, MTR_Rex_NOE, and MTR_Rex_MT, with additional B1-correction, as described in [14] utilizing both low power scans.
2. Lorentzian fit of the B1 = 0.6 µT spectra providing MT_const_ as described in [15].

*Asymmetry-based CEST*: This feature was obtained in line with recent consensus recommendations [24] using the identical snapshot readout described earlier and a presaturation as described by Zhou et al. (B_1_ = 2 µT, t_sat_ = 0.2 s, 95% duty cycle) over 16 offsets (± 4 to ±3ppm). APTw_asym_ was computed as Z(–3.5 ppm)–Z(3.5 ppm) [13]. Scanning time was 2:00 min/scan.

### Diffusion- and perfusion MRI

Single-shot echo-planar diffusion-weighted imaging (DWI) was performed with b-values of 0 and 1000 s/mm², and isotropic voxel sizes (2 × 2 × 2 mm³). ADC maps were calculated automatically by the scanner software from the acquired b-values following an FDA-approved protocol.

Dynamic susceptibility contrast (DSC) perfusion imaging was performed using gradient echo (GE) echo planar imaging (EPI) during the first pass of a gadolinium-based contrast bolus. A preload bolus (0.025 mmol of gadobutrol per kg of body weight) was administered 5 min before the DSC sequence to reduce T_1_-leakage effects. Subsequently, DSC acquisition was performed following a second bolus (0.05 mmol/kg), injected at 5 mL/s followed by 20 mL saline. Cerebral blood volume (CBV) maps were generated using the vendor software [25]. The CBV maps were normalized to contralateral normal appearing white matter (nCBV) in Matlab^®^ (v2024b, Mathworks) after coregistration (see below) [26].

### Tumor segmentation

Image coregistration was performed in the open-source image tool kit MITK (v2023.06). Whole tumor (WT) and contrast-enhancing tumor (CE) volumes were segmented in 3D on contrast-enhanced T1w and T2w-FLAIR images using custom Matlab^®^ (v2024b, Mathworks) software. In patients with residual contrast enhancement, WT comprised CE plus peritumoral hyperintensity on T2w. Segmentations were performed by an experienced radiologist with eight years of experience in neuroimaging (NK). Postoperative dispositions of blood breakdown products (e.g. methemoglobin and hemosiderin) were excluded by correlation with non-contrast-enhanced T1w and T2*w images.

### Statistical analysis

Receiver operator characteristic (ROC) analyses were performed to test for the associations of mean contrast values with therapy response. Dichotomized univariate Cox-Hazard regression analyses were performed to evaluate the associations of mean contrast values at baseline with PFS and OS. Dichotomized multivariate Cox-Hazard regression analyses were performed to evaluate the added benefit of CEST imaging to nCBV and ADC for PFS and OS prediction. Median contrast values were used for cohort dichotomization. Pearson correlation was used to assess the correlation of CEST contrasts with nCBV and ADC. Eight participants with unavailable perfusion imaging were censored in the uni- and multivariate Cox-Hazard regression analyses. All analyses were conducted in Matlab^®^ (v2024b, Mathworks) with a significance level set at *p* ≤ 0.05.

## Results

**Fig. 1:**
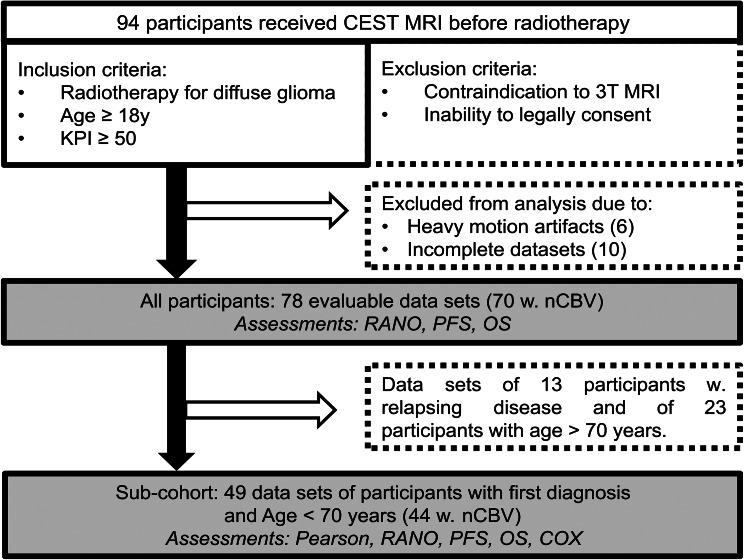
Flow Chart. 94 participants who underwent radiation therapy at the Department of Radiation Oncology of the University Hospital Heidelberg were enrolled in the study. Six participants were excluded from analysis due to heavy motion artifacts and ten due to incomplete data sets. Association of CEST contrast values with therapy response assessment according to the response assessment in neuro-oncology (RANO) criteria, progression-free survival (PFS) and overall survival (OS) were assessed. Analyses were performed in all 78 evaluable study participants and in a sub-cohort of 49 study participants with a first diagnosis of glioma and age ≤ 70 to control for confounders on mortality. Pearson correlation of CEST contrast values with normalized cerebral blood volume (nCBV) and apparent diffusion coefficient (ADC) maps, as well as added benefit of CEST contrasts to nCBV and ADC for PFS and OS prediction were analyzed in the sub-cohort

**Fig. 2:**
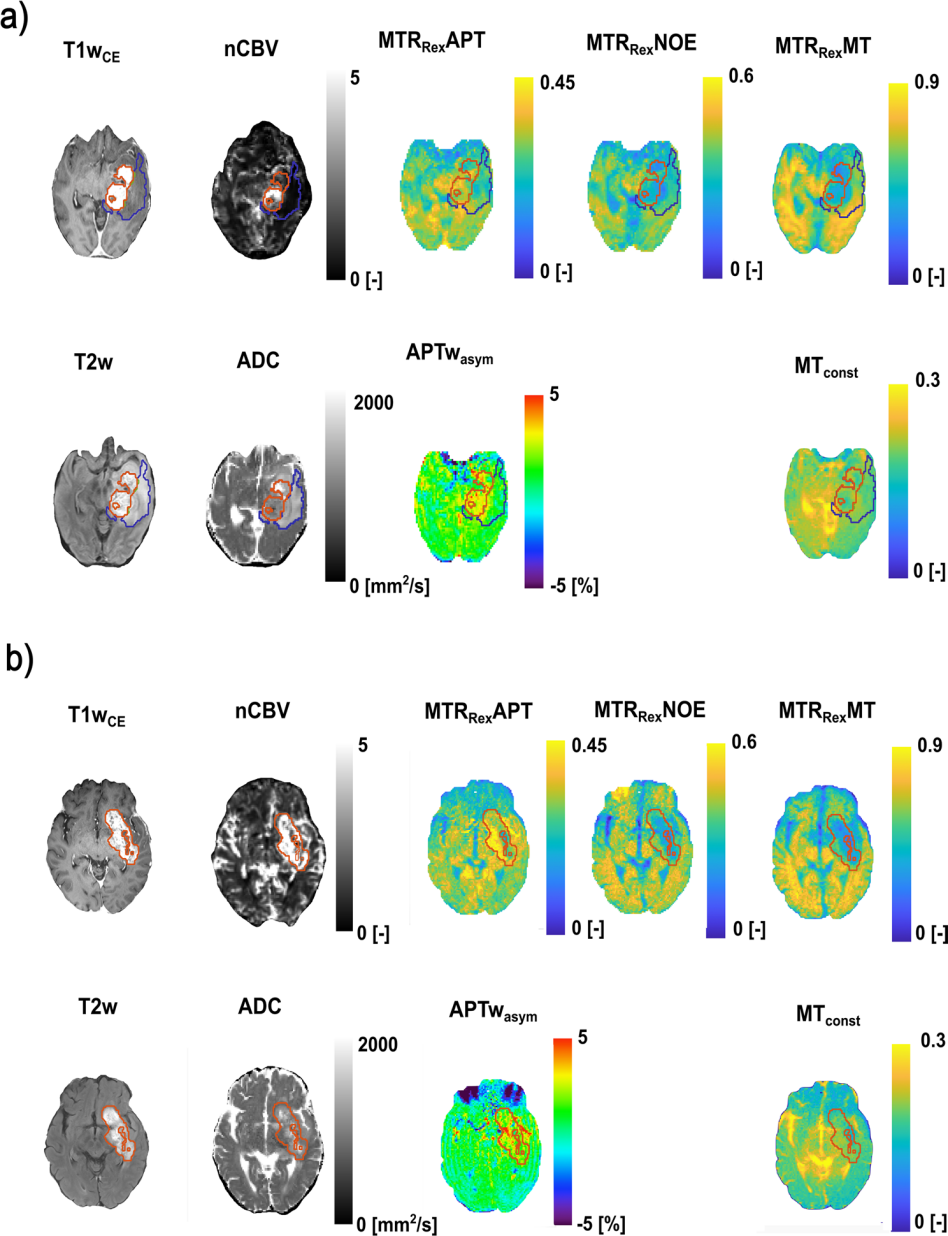
Exemplary contrast maps. Top rows (**a**) show exemplary contrast-enhanced T1-weighted (T1wCE)-, fluid-suppressed T2-weighted- (T2w), MTR_Rex_APT-, MTR_Rex_NOE-, MTR_Rex_MT-, APTw_asym_, MT_const_, normalized cerebral blood volume- (nCBV) and apparent diffusion coefficient (ADC) maps of a 58 year-old male study participant. The participant previously underwent debulking surgery for a first diagnosis of glioma, received combined radio- and chemotherapy (RT/CXT) following the baseline scan, and had not reached overall survival (OS) at a follow-up of 28.7 months at data cut-off (with a median OS of 11.58 months for the entire study cohort). Bottom rows (**b**) show respective contrast maps of a 64 year-old male study participant who previously underwent biopsy for a first diagnosis of glioma, received combined radio- and chemotherapy following the baseline scan, and had a shorter survival (2.1 months) compared to the cohort median. Contrast-enhancing tumor (CE) is indicated with red lining. Surrounding T2w-hyperintense tissue changes are indicated with blue lining. Whole tumor volume is composed of CE plus surrounding T2w-hyperintense tissue changes. [-] = Arbitrary units

Associations with therapy response, PFS and OS were assessed in all 78 evaluable participants, and in a sub-cohort of 49 participants with a first diagnosis of glioma and age ≤ 70 years to account for age and disease status (initial vs. relapsing disease) as important mortality confounders. Another reason for this sub-selection was the known impact of age and organic brain degeneration on CEST contrasts without direct links to tumor growth rates and brain infiltration [27, 28]. 70 was chosen as the cut-off, as age-related brain degeneration accelerates after the age of 65–70 and has been shown to be associated with changes in CEST signals [27, 29]. Furthermore, correlation of CEST contrasts with nCBV and ADC, as well as the added benefit of CEST contrasts to nCBV and ADC for PFS and OS prediction were assessed in the sub-cohort. A respective flow chart is depicted in Fig. 1. 64/78 participants overall, and 41/49 participants in the sub-cohort showed residual contrast enhancement on MRI (available CE). Exemplary CEST contrast maps are shown in Fig. 2.

### Imaging of the APTw_asym_, MTR_Rex_APT, MTR_Rex_NOE and MTR_Rex_MT is associated with therapy response

Therapy response assessment following completion of radiotherapy was available for 73/78 participants overall and for 47/49 participants in the sub-cohort (Table S1). 58/73 participants showed stable disease (SD), including twelve participants with initial pseudoprogression (PP). Fifteen study participants had progressive disease (PD). CE was present in 59/73 participants overall, and in 39/47 in the sub-cohort.

**Fig. 3:**
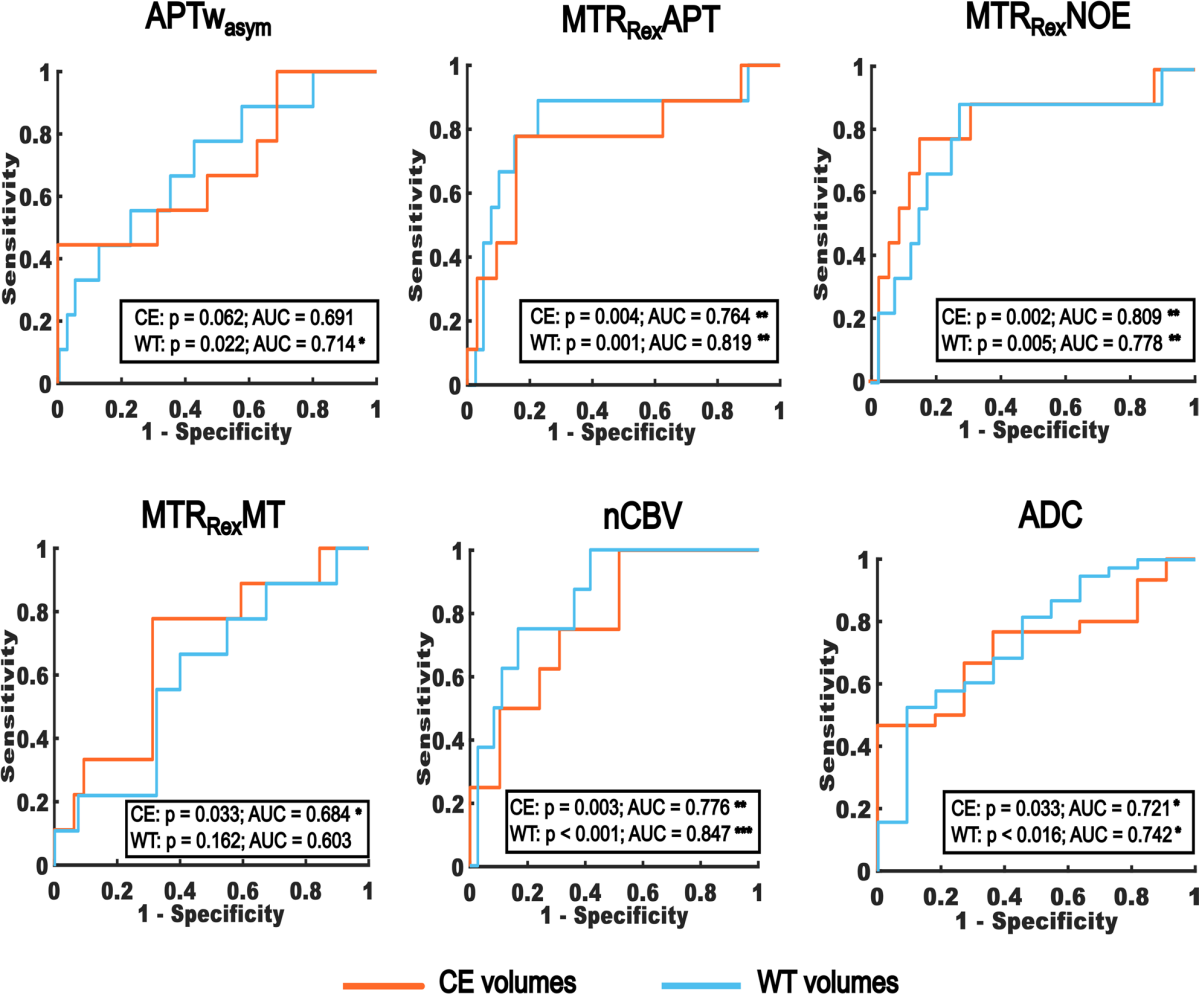
Association with therapy response. Receiving operator characteristic (ROC) plots showing significant (*p* ≤ 0.05) associations of mean values of CEST contrasts, normalized cerebral blood volume (nCBV) and apparent diffusion coefficient (ADC) at baseline MRI with therapy response according to the response assessment in neuro-oncology (RANO) criteria following completion of radiotherapy. Compared were participants with stable (SD) and progressive disease (PD). Shown are the results for 49 participants with an initial diagnosis of diffuse glioma and an age ≤ 70 years (sub-cohort). Residual contrast enhancement on MRI was present in 41/49 participants. CE = Contrast-enhancing tumor volume. WT = Whole tumor volume. *P* ≤ 0.05, ≤ 0.01 and ≤ 0.001 are indicated with “*”, “**” and “***” respectively

When comparing participants with SD (including PP) and PD in all evaluable participants, no association with therapy response was observed for any contrast. However, in the sub-cohort, the APTw_asym_ (WT: AUC = 0.714, *p* = 0.022*; CE: AUC = 0.691, *p* = 0.062) showed a weak to moderate association with therapy response for WT. Imaging of the MTR_Rex_APT (WT: AUC = 0.819, *p* = 0.001***; CE: AUC = 0.764, *p* = 0.004**), MTR_Rex_NOE (WT: AUC = 0.778, *p* = 0.005**; CE: AUC = 0.809, *p* = 0.002**), nCBV (WT: AUC = 0.847, *p* < 0.001***; CE: AUC = 0.776, *p* = 0.003**) and ADC (WT: AUC = 0.74, *p* = 0.016*; CE: AUC = 0.72, *p* = 0.033*) showed moderate associations with therapy response for WT and CE. The MTR_Rex_MT (WT: AUC = 0.603, *p* = 0.161; CE: AUC = 0.684, *p* = 0.033*) showed a weak association with response for CE. The MT_const_ showed no association with therapy response. Table 1 gives a summary of the results. Figure 3 illustrates the ROC plots for the sub-cohort.

**Table 1:** Association of imaging contrasts with therapy response. Results of the receiving operator characteristic (ROC) tests evaluating the association of CEST contrast values, normalized cerebral blood volume (nCBV) and apparent diffusion coefficient (ADC) at baseline with therapy response according the response assessment in neuro-oncology (RANO) criteria following completion of radiotherapy

Therapy response		All participants		Sub-cohort	
Tumor volume		WT	CE	WT	CE
APTw_asym_	P	0.448	0.099	0.022*	0.062
	AUC	0.656	0.618	**0.714**	0.691
MTR_REX_APT	P	0.633	0.548	0.001***	0.004**
	AUC	0.710	0.673	**0.819**	**0.764**
MTR_REX_NOE	P	0.249	0.940	0.005**	0.002**
	AUC	0.677	0.683	**0.778**	**0.809**
MTR_REX_MT	P	0.687	0.599	0.161	0.033*
	AUC	0.566	0.605	0.603	**0.684**
MT_const_	P	0.162	0.381	0.356	0.130
	AUC	0.521	0.567	0.517	0.576
nCBV	P	0.941	0.422	< 0.001***	0.003**
	AUC	0.741	0.755	**0.847**	**0.776**
ADC	P	0.344	0.355	0.016*	0.033*
	AUC	0.570	0.617	**0.742**	**0.721**
Participants (n)		78	64	49	41
Censored (n)		5	5	2	2

Given are levels of significance (P), area under the curves (AUC), best pair for sensitivity (BP sens) and best pair for specificity (BP spec). Furthermore, results for whole tumor (WT) and contrast-enhancing tumor volumes (CE) for all 78 evaluable study participants and a sub-cohort of 49 participants with a first diagnosis of glioma and age ≤ 70 years are shown. 64/78 participants overall, and 41/49 participants in the sub-cohort had residual contrast enhancement on MRI. Five and two participants were censored respectively due to missing follow-up image data. *P* ≤ 0.05, ≤ 0.01 and ≤ 0.001 is indicated with “*”, “**” and “***” respectively

### Imaging of the MTR_Rex_APT, MTR_Rex_NOE, MTR_Rex_MT, nCBV and ADC is associated with PFS

Median PFS for all participants was 6.04 months (95%-CI: 4.60; 10.45). Overall, the data of 25/78 participants (17/64 with available CE) was censored in the uni-variate Cox-Hazard regression analysis due to unavailable nCBV maps (*n* = 8 and 6, respectively), no progression at data cut-off (*n* = 16 and 10) and missing follow-up (*n* = 1 and 1). In the sub-cohort, the data of 17/49 (14/41) participants were censored for the same reasons (*n* = 5/4; 11/9; 1/1).

When all participants were analyzed, the ADC (WT: HR = 0.682, *p* = 0.178; CE: HR = 0.455, *p* = 0.014*) showed an association with PFS for CE volumes. However, none of the evaluated CEST contrasts, nor nCBV yielded significant results. Yet, in the sub-cohort, the MTR_Rex_APT (WT: HR = 1.35, *p* = 0.418; CE: HR = 2.736, *p* = 0.016*), and MTR_Rex_MT (WT: HR = 1.584, *p* = 0.209; CE: HR = 2.440, *p* = 0.028*) showed moderate associations with PFS for CE, whilst nCBV (WT: HR = 2.53, *p* = 0.013*; CE: HR = 1.73, *p* = 0.172) showed a moderate association with PFS for WT. The MTR_Rex_NOE (WT: HR = 3.750, *p* = 0.001***; CE: HR = 3.454, *p* = 0.003**) and ADC (WT: HR = 0.211, *p* = 0.001***; CE: HR = 0.179, *p* = 0.001***) showed strong associations with PFS for both, CE and WT. Again, imaging of the APTw_asym_ and MT_const_ was not associated with PFS.

**Fig. 4:**
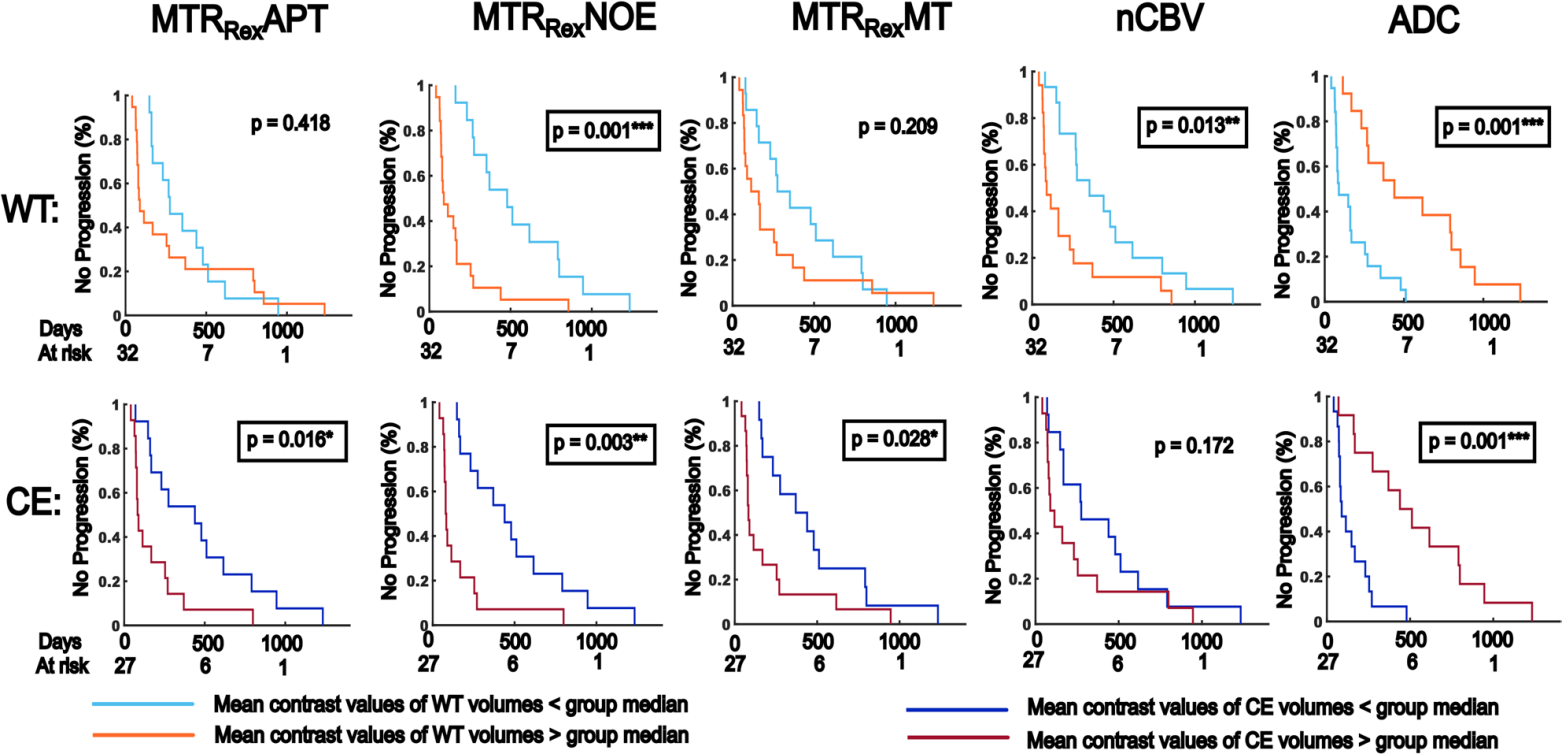
Association of mean contrast values with progression-free survival (PFS). Depicted are the Kaplan-Meier plots showing the results of dichotomized cox-hazard regression analyses testing for the association of mean CEST contrast values, normalized cerebral blood volume (nCBV) and apparent diffusion coefficient maps (ADC) with PFS in participants with a first diagnosis of diffuse glioma and age ≤ 70 years (sub-cohort). Data sets of five participants without perfusion imaging were censored. Group median contrast values were used for cohort dichotomization. The top row shows the results for whole tumor volumes (*n* = 44). The bottom row shows the results for contrast-enhancing tumor volumes (CE) in 37 participants with residual contrast enhancement on MRI. The curves show progression-free survival rates of participants with lower and higher mean contrast values compared to the cohort medians. *P* ≤ 0.05, ≤ 0.01 and 0.001 are indicated with “*”, “**” and “***” respectively

Table 2 gives a summary of the results. Figure 4 shows Kaplan-Meier plots illustrating the results for MTR_Rex_APT, MTR_Rex_NOE, MTR_Rex_MT, nCBV and ADC in the sub-cohort.

**Table 2:** Association with progression-free survival (PFS). Results of dichotomized Cox-Hazard regression analyses testing for the association of mean CEST contrast values, normalized cerebral blood volumes (nCBV) and apparent diffusion coefficients (ADC) at baseline before radiotherapy with PFS in all 78 evaluated study participants and in the sub-cohort of 49 study participants with a first diagnosis of glioma and age ≤ 70 years

Progression-free survival (PFS)		All participants		Sub-cohort	
Tumor volume:		WT	CE	WT	CE
APTw_asym_	P	0.461	0.531	0.887	0.700
	HR	1.235	1.207	0.949	1.166
	95% CI	0.704; 2.167	0.67; 2.173	0.46; 1.958	0.534; 2.544
	Median val. [%]	1.093	1.295	1.190	1.375
	PFS+	172.5	94	267	114
	PFS-	170	177	168	211
MTR_REX_APT	P	0.552	0.078	0.418	0.016*
	HR	1.182	1.722	1.350	**2.736**
	95% CI	0.682; 2.049	0.941; 3.15	0.653; 2.793	1.205; 6.211
	Median val. [-]	0.250	0.240	0.254	0.248
	PFS+	94	94	89	86
	PFS-	249	294.5	275	439
MTR_REX_NOE	P	0.362	0.149	0.001***	0.003**
	HR	1.293	1.557	**3.750**	**3.454**
	95% CI	0.744; 2.248	0.853; 2.844	1.717; 8.192	1.51; 7.903
	Median val. [-]	0.313	0.272	0.316	0.281
	PFS+	131	104	89	81
	PFS-	314	275	479	439
MTR_REX_MT	P	0.995	0.786	0.209	0.028*
	HR	0.998	1.085	1.584	**2.440**
	95% CI	0.577; 1.729	0.601; 1.959	0.772; 3.248	1.101; 5.407
	Median val. [-]	0.458	0.361	0.469	0.387
	PFS+	168	168	138	83
	PFS-	231	169	314	404.5
MT_const_	P	0.770	0.747	0.228	0.979
	HR	1.085	0.905	1.572	0.989
	95% CI	0.626; 1.881	0.495; 1.655	0.754; 3.276	0.438; 2.234
	Median val. [-]	0.165	0.165	0.167	0.163
	PFS+	184	168	168	141
	PFS-	165.5	200.5	249	270
nCBV	P	0.084	0.071	0.013*	0.172
	HR	1.634	1.733	**2.530**	1.730
	95% CI	0.937; 2.852	0.955; 3.143	1.212; 5.284	0.789; 3.795
	Median val. [-]	1.534	1.776	1.534	1.778
	PFS+	104	89	89	101.5
	PFS-	275	272.5	352	275
ADC	P	0.178	0.014*	0.001***	0.001***
	HR	0.682	**0.455**	**0.211**	**0.179**
	95% CI	0.391; 1.19	0.242; 0.854	0.086; 0.515	0.065; 0.494
	Median val. [mm^2^/s]	1203	1279.403	1174	1226
	PFS+	314	318	439	475
	PFS-	131	104	89	89
Participants (n)		78	64	49	41
Censored (n)		25	17	17	14

Residual contrast-enhancement on MRI was present in 64/78 and 41/49 participants respectively. 25 and 17 participants in all participants, as well as 17 and 14 participants in the sub-cohort were censored due to unavailable nCBV (*n* = 8, 6, 5 and 4), no progression at data cut-off (*n* = 16, 10, 11 and 9) and missing follow-up data (*n* = 1 both groups). Median contrast values were used for cohort dichotomization. Given are levels of significance (P), hazard ratios (HR), 95% confidence intervals (CI) of the HR, median contrast values, as well as PFS in days for participants with higher (PFS+) and lower (PFS-) contrast values compared to the cohort medians. WT = Whole tumor volume. CE = Contrast-enhancing tumor volume (CE). [-] = Arbitrary units. *P* ≤ 0.05, ≤ 0.01 and ≤ 0.001 are indicated with “*”, “**” and “***” respectively

### Imaging of the MTR_Rex_APT, MTR_Rex_NOE, MTR_Rex_MT and MT_const_ is associated with OS

Median OS was 11.58 months (95%-CI: 8.41; 14.36). Overall, the data of 37/78 participants (27/64 with available CE) was censored in the univariate Cox-Hazard regression analysis due to unavailable nCBV maps (*n* = 8 and 6, respectively), being alive at data cut-off (*n* = 26 and 18), and missing follow-up (*n* = 3 and 3). In the sub-cohort, the data of 25/49 (20/41) participants were censored for the same reasons (*n* = 5/4; 19/15; 1/1).

In all participants, the MTR_Rex_MT (WT: HR = 1.910, *p* = 0.050*; CE: 1.339, *p* = 0.392) and MT_const_ (WT: HR = 2.319, *p* = 0.018*; CE: HR = 1.968, *p* = 0.059) showed weak to moderate associations with OS for WT. The APTw_asym_, MTR_Rex_APT, MTR_Rex_NOE, nCBV and ADC on the other hand did not show any association with survival.

In the sub-cohort, the MTR_Rex_APT (WT: HR = 2.737, *p* = 0.026*; CE: HR = 2.889, *p* = 0.028*), MTR_Rex_NOE (WT: HR = 3.423, *p* = 0.012*; CE: HR = 2.963, *p* = 0.025*), MTR_Rex_MT (WT: HR = 3.134, *p* < 0.014*; CE: HR = 5.832, *p* = 0.003**) and MT_const_ (WT: HR = 4.401, *p* = 0.007**; CE: HR = 4.469, *p* = 0.008**) showed moderate to strong associations with OS for WT and CE volumes. The nCBV (WT: HR = 1.922, *p* = 0.145; CE: HR = 3.643, *p* = 0.007**) also showed a strong association with OS for CE volumes. The APTw_asym_ and ADC did not show any association with OS again.

**Fig. 5:**
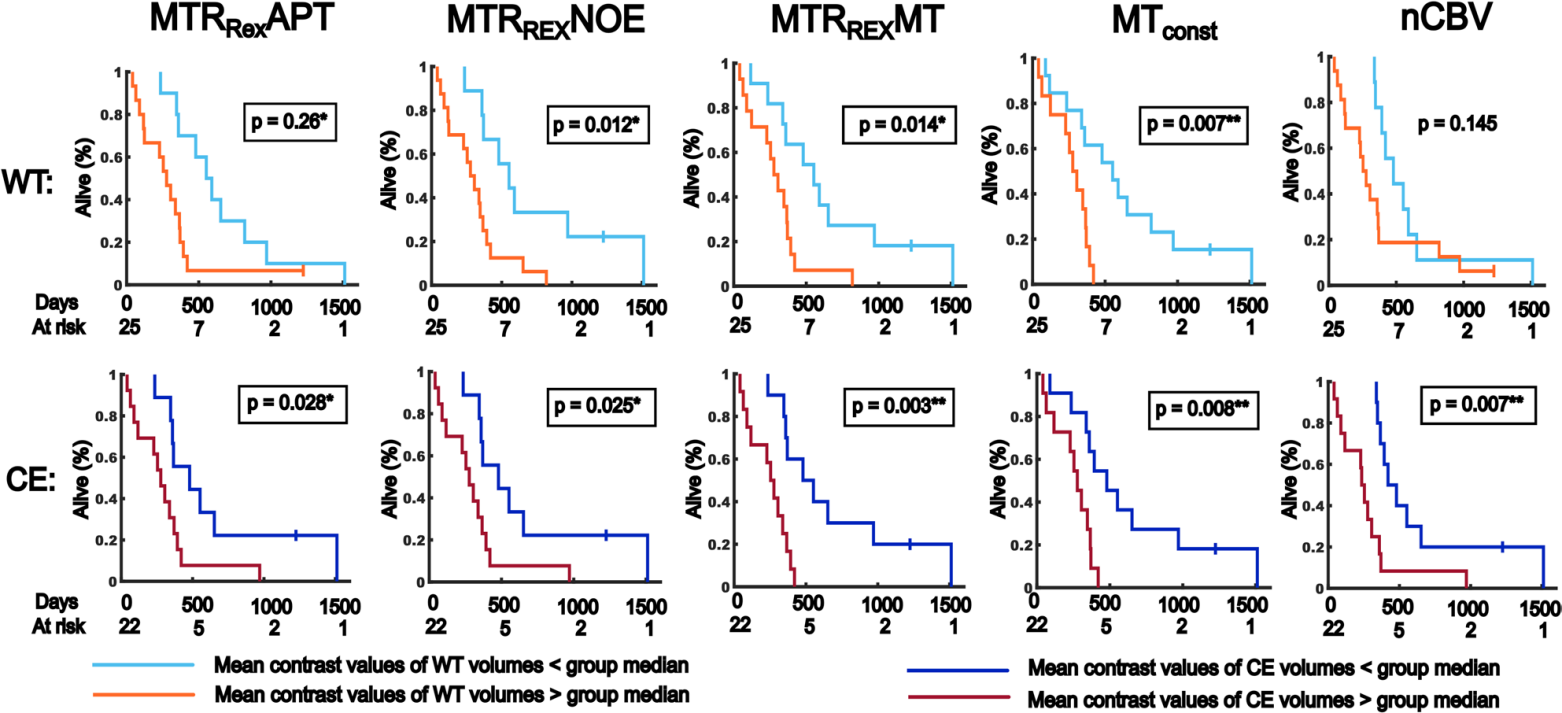
Association of mean contrast values with overall survival (OS). Depicted are the Kaplan-Meier plots showing the results of dichotomized cox-hazard regression analyses testing for the association of mean CEST contrast values, normalized cerebral blood volume (nCBV) and apparent diffusion coefficient maps (ADC) with OS in participants with a first diagnosis of diffuse glioma and age ≤ 70 years (sub-cohort). Data sets of five participants without perfusion imaging were censored. Group median contrast values were used for cohort dichotomization. The top row shows the results for whole tumor volumes (*n* = 44). The bottom row shows the results for contrast-enhancing tumor volumes (CE) in 37 participants with residual contrast enhancement on MRI. The curves show survival rates of participants with lower and higher mean contrast values compared to the cohort medians. *P* ≤ 0.05, ≤ 0.01 and 0.001 are indicated with “*”, “**” and “***” respectively

Table 3 gives a summary of the results. Figure 5 shows Kaplan-Meier plots illustrating the results for MTR_Rex_APT, MTR_Rex_NOE, MTR_Rex_MT, MT_const_ and nCBV in the sub-cohort.

**Table 3:** Results of dichotomized Cox-Hazard regression analyses testing for the association of mean CEST contrast values, normalized cerebral blood volumes (nCBV) and apparent diffusion coefficient maps (ADC) at baseline before radiotherapy with overall survival (OS) in all 78 evaluated study participants and in the sub-cohort of 49 study participants with a first diagnosis of glioma and age ≤ 70 years

Overall survival (OS)		All participants		Sub-cohort	
Tumor volume:		WT	CE	WT	CE
APTw_asym_	P	0.384	0.322	0.532	0.619
	HR	1.325	1.401	1.303	1.254
	95% CI	0.703; 2.496	0.719; 2.731	0.568; 2.988	0.514; 3.062
	Median val. [%]	1.093	1.295	1.190	1.375
	OS+	321.5	223	338	321.5
	OS-	383	368	364.5	368
MTR_Rex_APT	P	0.113	0.127	0.026*	0.028*
	HR	1.680	1.694	**2.737**	**2.889**
	95% CI	0.885; 3.189	0.86; 3.336	1.131; 6.624	1.119; 7.464
	Median val. [-]	0.250	0.240	0.254	0.248
	OS+	291	277	277	277
	OS-	459	441	571	479
MTR_Rex_NOE	P	0.087	0.121	0.012*	0.025*
	HR	1.755	1.703	**3.423**	**2.963**
	95% CI	0.921; 3.344	0.868; 3.338	1.308; 8.96	1.147; 7.651
	Median val. [-]	0.313	0.272	0.316	0.281
	OS+	315	305	291	277
	OS-	441	359	552	479
MTR_Rex_MT	P	0.050*	0.392	0.014*	0.003**
	HR	**1.910**	1.339	**3.134**	**5.832**
	95% CI	1.001; 3.645	0.686; 2.611	1.255; 7.83	1.82; 18.687
	Median val. [-]	0.458	0.361	0.469	0.387
	OS+	310	312.5	291	265.5
	OS-	460	352.5	552	515.5
MT_const_	P	0.018*	0.059	0.007**	0.008**
	HR	**2.319**	1.968	**4.401**	**4.469**
	95% CI	1.155; 4.655	0.974; 3.977	1.499; 12.922	1.489; 13.412
	Median val. [-]	0.165	0.165	0.167	0.163
	OS+	315	310	291	277
	OS-	441	376	552	479
nCBV	P	0.311	0.333	0.145	0.007**
	HR	1.407	1.396	1.922	**3.643**
	95% CI	0.726; 2.726	0.71; 2.741	0.798; 4.628	1.432; 9.267
	Median val. [-]	1.534	1.776	1.534	1.778
	OS+	291	265.5	265.5	244.5
	OS-	408.5	395	479	449.5
ADC	P	0.767	0.309	0.448	0.091
	HR	0.907	0.709	0.696	0.413
	95% CI	0.477; 1.725	0.365; 1.376	0.273; 1.774	0.148; 1.153
	Median val. [mm^2^/s]	1203	1279.403	1174	1226
	OS+	370	370	370	370
	OS-	315	277	321.5	305
Participants (n)		78	64	49	41
Censored (n)		37	27	25	20

Residual contrast-enhancement on MRI was present in 64/78 and 41/49 participants respectively. Thirty-seven and 27 participants overall, as well as 25 and 20 participants in the subcohort, were censored due to unavailable nCBV maps (*n* = 8, 6, 5 and 4), being alive at data cut-off (*n* = 26, 18, 19 and 15) and missing follow-up data (*n* = 3, 3, 1 and 1). Median contrast values were used for cohort dichotomization. Given are levels of significance (P), hazard ratios (HR), 95% confidence intervals (CI) of the HR, median contrast values, as well as OS in days for participants with higher (OS+) and lower (OS-) contrast values compared to the cohort medians. WT = Whole tumor volume. CE = Contrast-enhancing tumor volume (CE). [-] = Arbitrary units. *P* ≤ 0.05, ≤ 0.01 and ≤ 0.001 are indicated with “*”, “**” and “***” respectively

### Added benefit of the MTR_Rex_MT and MT_const_ for OS prediction

These analyses were performed in the sub-cohort of 49 participants. Combinations of investigated contrasts and tumor volumes were selected based on the results above. The numbers of included and censored participants are the same as above.

**Fig. 6:**
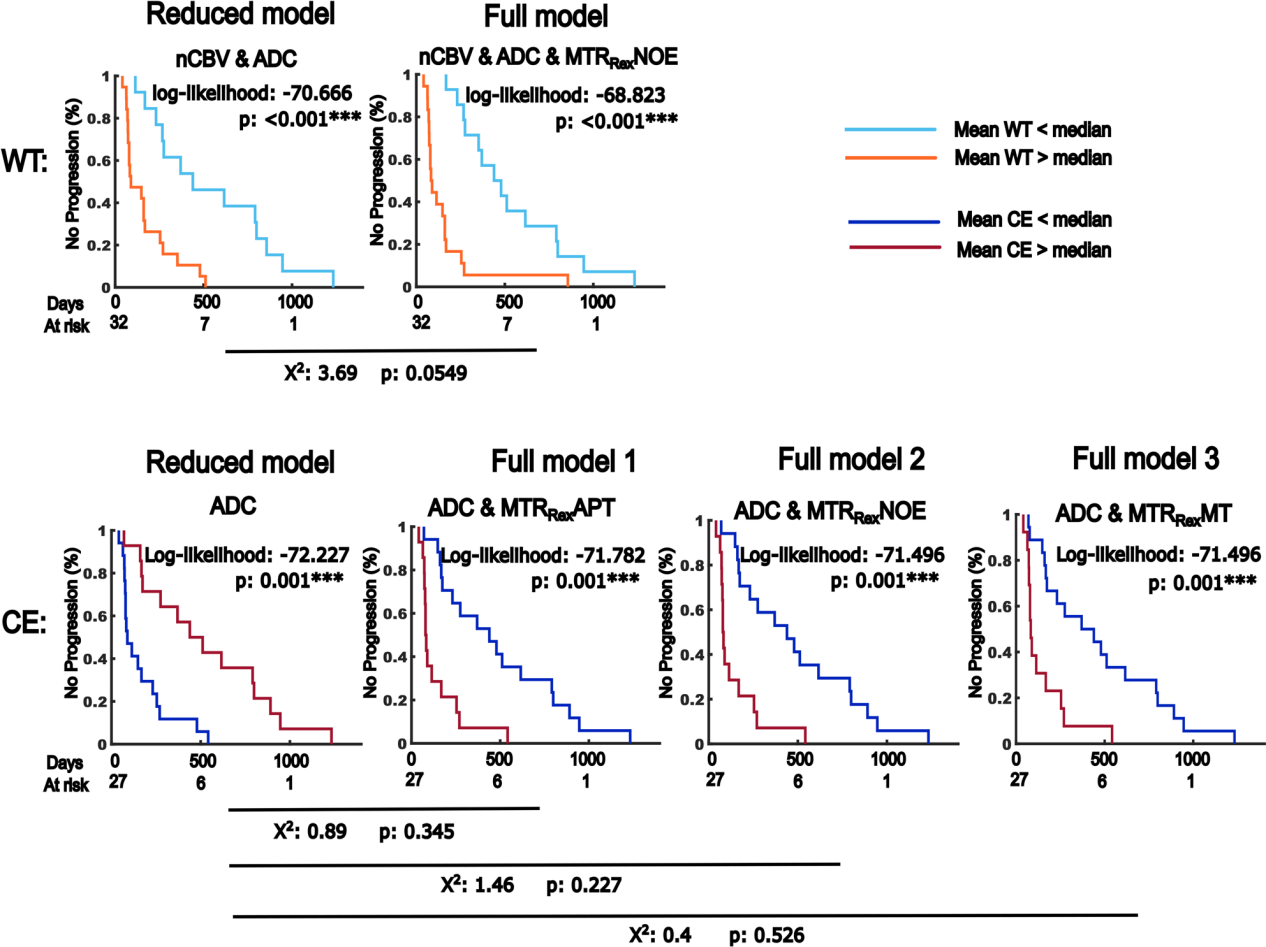
Added value of CEST contrasts to normalized cerebral blood volume (nCBV) and apparent diffusion coefficient maps (ADC) for the prediction of progression-free survival (PFS). Shown are the Kaplan-Meier graphs of reduced and full models depicting survival rates of participants with high and low risk on multivariate dichotomized cox-hazard regression analyses. Contrast combinations were selected based on the results of the univariate cox-hazard regression analysis for PFS prediction. Combination of median contrast values were used for cohort dichotomization. Given are the log-likelihood- and p-values for reduced and full models as well as the results of the likelihood ratio tests (χ2) evaluating performance differences between the models with respective p-values. The analysis was conducted in participants with a first diagnosis of diffuse glioma and age ≤ 70 years (sub-cohort). The data of five participants without available perfusion data was censored in this analysis. 44 participants were available for assessment of whole tumor volume (WT) ROIs and 37 participants with residual contrast enhancement on MRI for assessment of contrast-enhancing tumor volumes (CE). *P* ≤ 0.05, ≤ 0.01 and ≤ 0.001 are indicated with “*”, “**” and “***” respectively

For PFS prediction, the added benefit of the ADC to nCBV was assessed in WT volumes first. Subsequently, the added benefit of MTR_Rex_NOE to nCBV and ADC in WT volumes, as well as of the MTR_Rex_APT, MTR_Rex_NOE and MTR_Rex_MT to ADC in CE volumes was assessed. ADC maps showed added benefit to nCBV for PFS prediction in WT volumes, with χ^2^ = 15.94 and *p* < 0.001*** in the likelihood ratio test. The addition of the MTR_Rex_NOE improved the model-fit of the multivariate Cox-Hazard regression analyses marginally, without being statistically significant (χ^2^ = 3.69, *p* = 0.055). The addition of the MTR_Rex_APT, MTR_Rex_NOE and MTR_Rex_MT to ADC did not improve the model-fit when CE volumes were assessed. Table 4; Fig. 6 yield a summary and a graphical depiction of the results.

**Table 4:** Results of dichotomized Cox-Hazard regression analyses testing for the association of mean CEST contrast values, normalized cerebral blood volumes (nCBV) and apparent diffusion coefficient maps (ADC) at baseline before radiotherapy with overall survival (OS) in all 78 evaluated study participants and in the sub-cohort of 49 study participants with a first diagnosis of glioma and age ≤ 70 years

Dichotomized multivariate cox-hazard regression analysis for PFS and overall survival prediction								
**ROI**	**Full model**	**Log-like-lihood**	**P-value**	**Reduced model**	**Log-like-lihood**	**P-value**	**Like-lihood ratio test (χ**^**2**^)	**P (χ** ^**2**^ **)**
**Prediction of progression-free survival**								
WT	ADC	**-70.666**	< 0.001***	**nCBV**	**-78.635**	0.0132*	**15.94**	< 0.001***
	nCBV							
WT	NOE_MTRRex_	**-68.823**	< 0.001***		**-70.666**	< 0.001***	**3.69**	**0.0549**
	ADC			**ADC**				
	nCBV			**nCBV**				
CE	APT_MTRRex_	**-71.782**	0.002**	**ADC**	**-72.227**	0.001***	0.89	0.3454
	ADC							
CE	NOE_MTRRex_	**-71.496**	0.001***	**ADC**	**-72.227**	0.001***	1.46	0.2266
	ADC							
CE	MT_MTRRex_	**-72.024**	0.002**	**ADC**	**-72.227**	0.001***	0.4	0.5245
	ADC							
**Prediction of overall survival**								
CE	APT_MTRRex_	**-43.727**	0.0174*	**nCBV**	**-44.023**	0.006**	0.59	0.4415
	nCBV							
CE	NOE_MTRRex_	**-43.067**	0.009**	**nCBV**	**-44.023**	0.006**	1.91	0.1667
	nCBV							
CE	MT_MTRRex_	**-37.17**	< 0.001***	**nCBV**	**-44.023**	0.006**	**13.71**	< 0.001***
	nCBV							
CE	MT_const_	**-41.212**	0.001***	**nCBV**	**-44.023**	0.006**	**5.62**	0.0177*
	nCBV							

Residual contrast-enhancement on MRI was present in 64/78 and 41/49 participants respectively. Thirty-seven and 27 participants over all, as well as 25 and 20 participants in the subcohort, were censored due to unavailable nCBV maps (*n* = 8, 6, 5 and 4), being alive at data cut-off (*n* = 26, 18, 19 and 15) and missing follow-up data (*n* = 3, 3, 1 and 1). Median contrast values were used for cohort dichotomization. Given are levels of significance (P), hazard ratios (HR), 95% confidence intervals (CI) of the HR, median contrast values, as well as OS in days for participants with higher (OS+) and lower (OS-) contrast values compared to the cohort medians. WT = Whole tumor volume. CE = Contrast-enhancing tumor volume (CE). [-] = Arbitrary units. *P* ≤ 0.05, ≤ 0.01 and ≤ 0.001 are indicated with “*”, “**” and “***” respectively

**Fig. 7:**
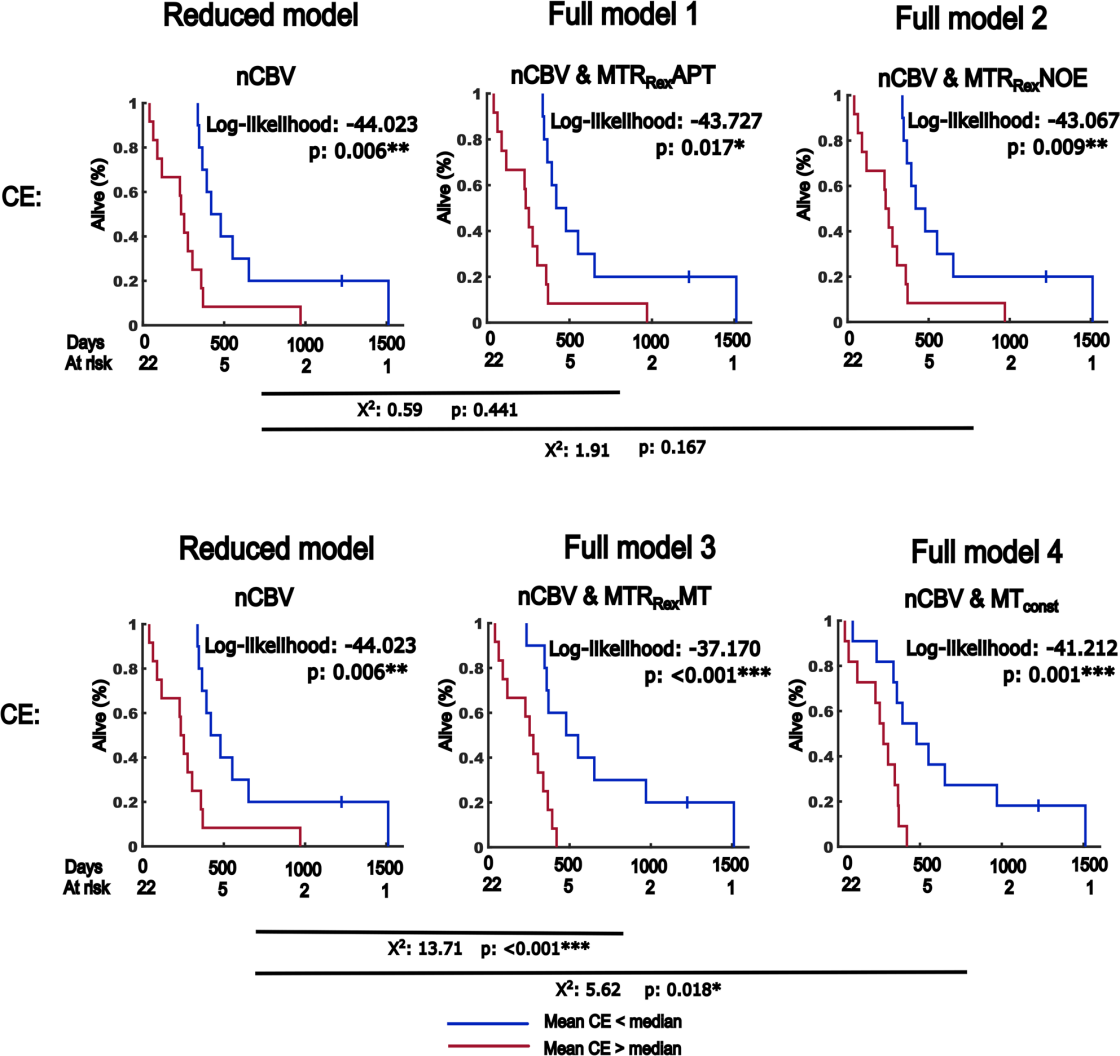
Added value of CEST contrasts to normalized cerebral blood volume (nCBV) for the prediction of overall survival. Shown are the Kaplan-Meier graphs of reduced and full models depicting survival rates of participants with high and low risk on multivariate dichotomized cox-hazard regression analyses. Contrast combinations were selected based on the results of the univariate cox-hazard regression analysis for OS prediction. Combinations of median contrast values were used for cohort dichotomization. Given are the log-likelihood- and p-values for reduced and full models as well as the results of the likelihood ratio tests (χ2) evaluating performance differences between the models with respective p-values. The analysis was conducted in participants with a first diagnosis of diffuse glioma and age ≤ 70 years (sub-cohort). The data of five participants without available perfusion data was censored in this analysis. 44 participants were available for assessment of whole tumor volume (WT) ROIs and 37 participants with residual contrast enhancement on MRI for assessment of contrast-enhancing tumor volumes (CE). *P* ≤ 0.05, ≤ 0.01 and ≤ 0.001 are indicated with “*”, “**” and “***” respectively

For OS prediction, the added benefit of the MTR_Rex_APT, MTR_Rex_NOE, MTR_Rex_MT and MT_const_ to nCBV was assessed in CE volumes. The addition of MTR_Rex_MT (χ^2^ = 13.71, *p* < 0.001***) and MT_const_ (χ^2^ = 5.62, *p* = 0.018*) to nCBV in CE volumes improved the model-fit. However, no added benefit of the MTR_Rex_APT and MTR_Rex_NOE was observed in CE volumes. The results are summarized and shown in Table 4; Fig. 7.

The MTR_Rex_APT showed the strongest Pearson correlation with nCBV (*r* = 0.75, *p* < 0.001***), observed for CE volumes. The MTR_Rex_NOE showed the strongest inverse correlation with ADC, also observed for CE volumes (*r* = − 0.76, *p* < 0.001***). See Fig. S1 for results.

## Discussion

In this prospective study, the predictive potential and added benefit of different APT-, rNOE and ssMT-weighted CEST contrasts on therapy outcome were comparatively assessed in a cohort of 78 study participants with diffuse glioma following surgery at baseline before radiotherapy at a clinical field strength of 3 T [13–15]. Imaging of the MTR_Rex_APT, MTR_Rex_NOE and MTR_Rex_MT showed moderate to strong associations with therapy response, PFS and OS, whilst the MT_const_ and APTw_asym_ were only associated with OS and therapy response, respectively. Only the MTR_Rex_MT and MT_const_ demonstrated added benefit to clinically-established nCBV imaging for OS prediction.

The current results obtained in patients with glioma following surgery align with prior reports demonstrating the potential of relaxation-compensated Lorentzian-fit-based APT imaging (MTR_Rex_APT) for PFS and OS prediction in patients with newly diagnosed gliomas [30]. The findings also reinforce previous studies from Regnery et al. at 7 T and of Mehrabian et al. at 3 T. These studies showed that Lorentzian-fit-based imaging of the rNOE without relaxation compensation was associated with therapy response and disease progression before the start of radiotherapy [15, 31]. The current study shows that also imaging of the rNOE using the relaxation compensated metric (MTR_Rex_NOE) showed ability to predict outcome when acquired after surgery and before radiotherapy. While Mehrabian et al. observed an association of ssMT imaging without relaxation compensation (MT_const_) with therapy response, this study found only an association of the MT_const_ with OS [15]. Furthermore, while Joo et al. reported a strong association of asymmetry-based APT imaging (APTw_asym_) with PFS and OS in patients with newly diagnosed glioma prior to resection [32], no such association of the APTw_asym_ with survival was observed in this study cohort of patients following surgery. Two other studies observed significant associations of the MT_const_ and APTw_asym_ with therapy response and survival in the early post-treatment interval that were not detected at baseline before radiotherapy in this study [16, 17]. Lastly, Joo et al. reported added benefit of the APTw_asym_ for outcome prediction to clinical features (e.g. IDH-mutation, patient age and KPS etc.) whilst Liu et al. and Park et al. reported the added benefit of the APTw_asym_ to nCBV and ADC imaging for the differentiation of recurrent tumor and radiation necrosis after radiotherapy [32, 34–36]. In the current study, added benefit of MT-weighted MTR_Rex_MT and MT_const_ imaging to nCBV was observed for OS prediction, when CE tumor volumes were evaluated. Model improvement that barely missed the significance level was also observed for PFS prediction when the MTR_Rex_NOE of WT volumes was added to ADC and nCBV imaging.

The APTw_asym_ is the most widely studied CEST MRI contrast and was initially proposed by Zhou et al. in 2003 [37]. This imaging method exploits the asymmetry of contributing peaks to the *Z*-spectrum up- and down-field from the water resonance to extract the amide peak at 3.5 ppm [37]. Consequently, the resulting APT-weighted contrast (APTw_asym_) has additional contributions from rNOE- and ssMT effects [8, 10, 12]. Subsequently, other groups applied multi-Lorentzian models to fit the individual APT, rNOE and ssMT peaks of the *Z*-spectrum [8, 10]. Yet, these contrasts still have spillover contributions from direct water saturation and signals from overlapping pools (e.g. MT_const_) [12, 15]. Therefore, Zaiss et al. suggested relaxation compensation of Lorentz-fitted CEST imaging, introducing an inverse metric to compensate for direct water saturation (MTR_Rex_APT, MTR_Rex_NOE and MTR_Rex_MT) [12]. This approach was later optimized for the application at 3T by Goerke et al. [14].

In conjunction with the outlined findings from other authors, the results of this study indicate that relaxation-compensated Lorentz-fit-based CEST contrasts of the APT, rNOE and ssMT may harbor higher prognostic value for outcome prediction in patients with glioma at baseline before radiotherapy. In the post-radiotherapy treatment monitoring of patients with glioma, APTw_asym_, however, demonstrated superior performance compared to the relaxation-compensated metric in predicting outcome, both in terms of PFS and OS [16, 17]. This differential performance of the individual metrics at different time points warrants further investigation.

The observed relatively strong correlation of the MTR_Rex_APT with nCBV could reflect the dependence of the predictive value of the MTR_Rex_APT on tumor characteristics related to perfusion. Possible underlying causes could be the high amide proton density of hemoglobin, alterations of the pH in tumor tissue affecting the proton exchange rate, increased cellular density in hyperperfused areas, or an overlap of such effects [8, 10]. These suggested interdependencies may furthermore be influenced by IDH mutation status, which is known to be prognostically important and has previously been associated with CEST-derived metrics [38]. This is due to its negative association with nCBV and its multifold effects on the metabolism of diffuse gliomas. The strong inverse correlation of the MTR_Rex_NOE and MTR_Rex_MT with the ADC could be linked to a dilution of protons bound in aliphatic and semi-solid macromolecules through tumor-associated edema. However, the fact that only the MTR_Rex_MT and MT_const_ added benefit to nCBV for OS prediction emphasizes the independent prognostic value of ssMT imaging in this cohort. The difference between the higher prognostic value of the MTR_Rex_APT, MTR_Rex_NOE and MTR_Rex_MT in the current study and the previously reported better performance of the APTw_asym_ and MT_const_ in the early post-treatment interval, might be related to prognostically relevant radiation-induced tissue alterations. These alterations might be more sensitively captured by APTw_asym_ and MT_const_ [16, 17]. However, this presumption requires further investigation.

The relatively small cohort size, heterogeneous patient profiles (see Table S1) and the limited follow-up were the most important limitations of this study. Furthermore, significant results were mostly obtained in a sub-cohort of 49 participants in which patients with an age > 70 years and relapsing disease were excluded. This observational difference might be linked to confounders related to mortality and metabolic changes of the brain parenchyma associated with degeneration [27, 28]. Nevertheless, statistically meaningful and significant results were obtained that further advance our understanding of the different CEST metrics and will motivate forthcoming studies. Furthermore, CE and WT did not distinguish postoperative blood-brain-barrier disruption from ischemia or granulation tissue versus viable tumor. Yet, as the study focused on evaluating CEST contrast for facilitated prognostication after surgery and before radiotherapy, this distinction was intentionally not pursued. Furthermore, even though the long scanning time (approx. 20 min for CEST data acquisition) likely prevents the clinical adaptation of the very imaging protocol applied in this study, protocols for faster acquisition of relaxation-compensated CEST contrasts have been developed [39].

## Conclusion

This study demonstrates the value of relaxation-compensated CEST imaging of the APT, rNOE and ssMT and APTw_asym_ for the prediction of therapy response and outcome of patients with diffuse glioma following surgery at baseline before radiotherapy at 3T. Furthermore, relaxation-compensated CEST metrics and the ssMT-weighted CEST contrasts showed added value for survival prediction compared to perfusion imaging. The results of this study further enhance our understanding of the predictive potential of CEST imaging in patients with glioma, particularly with respect to the differences between the individual CEST metrics, and may encourage the inclusion of CEST MRI as an imaging biomarker alongside perfusion- and diffusion-weighted imaging in brain tumor protocols.

## Supplementary Information

Below is the link to the electronic supplementary material.

Supplementary Material 1

## Data Availability

The datasets used and/or analyzed during the current study are available from the corresponding author upon reasonable request.

## Abbreviations

ADC: Apparent Diffusion Coefficient
APT: Amide Proton Transfer
APTw: Amide Proton Transfer–weighted
APTw_asym_: APTw magnetization transfer ratio asymmetry
AUC: Area Under the Curve
nCBV: Normalized Cerebral Blood Volume
CE: Contrast-enhancing Tumor
CEST: Chemical Exchange Saturation Transfer
CI: Confidence Interval
CNS: Central Nervous System
DWI: Diffusion-Weighted Imaging
FLAIR: Fluid-Attenuated Inversion Recovery
HR: Hazard Ratio
IDH: Isocitrate Dehydrogenase
LOH1p19q: Loss of Heterozygosity of chromosome arms 1p and 19q
KPS: Karnofsky Performance Status
MRI: Magnetic Resonance Imaging
MTR_Rex_: Relaxation-compensated Magnetization Transfer Ratio
MTR_Rex_APT: APT-weighted Relaxation-compensated Magnetization Transfer Ratio
MTR_Rex_MT: ssMT-weighted Relaxation-compensated Magnetization Transfer Ratio
MTR_Rex_NOE: rNOE-weighted Relaxation-compensated Magnetization Transfer Ratio
MT_const_: ssMT-weighted Constant Magnetization Transfer
rNOE: relayed Nuclear Overhauser Effect
OS: Overall Survival
PD: Progressive Disease
PET: Positron Emission Tomography
PFS: Progression-Free Survival
PP: Pseudoprogression
RANO: Response Assessment in Neuro-Oncology
RF: Radiofrequency
ROC: Receiver Operating Characteristic
ROI: Region of Interest
SD: Stable Disease
ssMT: Semi-solid Magnetization Transfer
T: Tesla
T1w: T1-weighted
T2w: T2-weighted
TE: Echo Time
TR: Repetition Time
WASABI: Water Shift And B1 mapping
WHO: World Health Organization
WT: Whole Tumor

## References

[1] Ostrom QT, et al. CBTRUS statistical report: primary brain and other central nervous system tumors diagnosed in the United States in 2015–2019. Neurooncology. 2022;24(Supplement5):v1–95. 10.1093/neuonc/noac202 PMC9533228 36196752

[2] Weller M, et al. EANO guidelines on the diagnosis and treatment of diffuse gliomas of adulthood. Nat Rev Clin Oncol. 2021;18(3):170–86. 33293629 10.1038/s41571-020-00447-z PMC7904519

[3] Louis DN, et al. The 2021 WHO Classification of Tumors of the Central Nervous System: a summary. Neuro Oncol. 2021;23(8):1231–51. 34185076 10.1093/neuonc/noab106 PMC8328013

[4] von Doeberitz K. Changing paradigms in oncology: Toward noncytotoxic treatments for advanced gliomas. Int J Cancer. 2022;151(9):1431–46. 35603902 10.1002/ijc.34131 PMC9474618

[5] Henriksen OM, et al. High-Grade Glioma Treatment Response Monitoring Biomarkers: A Position Statement on the Evidence Supporting the Use of Advanced MRI Techniques in the Clinic, and the Latest Bench-to-Bedside Developments. Part 1: Perfusion and Diffusion Techniques. Front Oncol. 2022;12:810263. 35359414 10.3389/fonc.2022.810263 PMC8961422

[6] De Marco R, et al. A systematic review of amino acid PET imaging in adult-type high-grade glioma surgery: a neurosurgeon’s perspective. Cancers (Basel). 2022;15(1). 10.3390/cancers15010090 PMC9817716 36612085

[7] van Mossel S, et al. A Systematic Literature Review of Modelling Approaches to Evaluate the Cost Effectiveness of PET/CT for Therapy Response Monitoring in Oncology. PharmacoEconomics. 2025;43(2):133–51. 39488797 10.1007/s40273-024-01447-y PMC11782410

[8] van Zijl PCM, et al. Magnetization Transfer Contrast and Chemical Exchange Saturation Transfer MRI. Features and analysis of the field-dependent saturation spectrum. NeuroImage. 2018;168:222–41. 28435103 10.1016/j.neuroimage.2017.04.045 PMC5650949

[9] von Doeberitz K. Chemical exchange saturation transfer (CEST) Magnetic resonance imaging in diagnostic oncology. Radiologe. 2021;61:43–51. 33337509 10.1007/s00117-020-00786-z

[10] Wu B, et al. An overview of CEST MRI for non-MR physicists. EJNMMI Phys. 2016;3(1):19. 27562024 10.1186/s40658-016-0155-2 PMC4999387

[11] Deng HZ, et al. Advances in diffuse glioma assessment: preoperative and postoperative applications of chemical exchange saturation transfer. Front Neurosci. 2024;18:1424316. 39148521 10.3389/fnins.2024.1424316 PMC11325484

[12] Zaiss M, et al. Relaxation-compensated CEST-MRI of the human brain at 7T: Unbiased insight into NOE and amide signal changes in human glioblastoma. NeuroImage. 2015;112:180–8. 25727379 10.1016/j.neuroimage.2015.02.040

[13] Zhou J, et al. APT-weighted MRI: Techniques, current neuro applications, and challenging issues. J Magn Reson Imaging. 2019;50(2):347–64. 30663162 10.1002/jmri.26645 PMC6625919

[14] Goerke S, et al. Relaxation-compensated APT and rNOE CEST-MRI of human brain tumors at 3 T. Magn Reson Med. 2019;82(2):622–32. 30927313 10.1002/mrm.27751

[15] Mehrabian H, et al. Evaluation of Glioblastoma Response to Therapy With Chemical Exchange Saturation Transfer. Int J Radiat Oncol Biol Phys. 2018;101(3):713–23. 29893279 10.1016/j.ijrobp.2018.03.057

[16] Kroh F, et al. Semi-solid MT and APTw CEST-MRI predict clinical outcome of patients with glioma early after radiotherapy. Magn Reson Med. 2023;90(4):1569–81. 37317562 10.1002/mrm.29746

[17] von Doeberitz K. CEST imaging of the APT and ssMT predict the overall survival of patients with glioma at the first follow-up after completion of radiotherapy at 3T. Radiother Oncol. 2023;184:109694. 37150450 10.1016/j.radonc.2023.109694

[18] von Doeberitz K. N. Post-surgical depositions of blood products are no major confounder for the diagnostic and prognostic performance of CEST MRI in patients with glioma. Biomedicines. 2023;11(9). 10.3390/biomedicines11092348 PMC10525358 37760790

[19] Wen PY, et al. RANO 2.0: Update to the Response Assessment in Neuro-Oncology Criteria for High- and Low-Grade Gliomas in Adults. J Clin Oncol. 2023;41(33):5187–99. 37774317 10.1200/JCO.23.01059 PMC10860967

[20] Louis DN, et al. The 2016 World Health Organization classification of tumors of the central nervous system: a summary. Acta Neuropathol. 2016;131(6):803–20. 27157931 10.1007/s00401-016-1545-1

[21] Zaiss M, Ehses P, Scheffler K. Optimizing spiral-centric-reordered gradient echo acquisition for fast and robust 3D CEST MRI at 9.4 T. NMR Biomed. 2018;31(4):e3879. 29372571 10.1002/nbm.3879

[22] Schuenke P, et al. Simultaneous mapping of water shift and B1 (WASABI)-Application to field-Inhomogeneity correction of CEST MRI data. Magn Reson Med. 2017;77(2):571–80. 26857219 10.1002/mrm.26133

[23] Breitling J, et al. Adaptive denoising for chemical exchange saturation transfer MR imaging. NMR Biomed. 2019;32(11):e4133. 31361064 10.1002/nbm.4133

[24] Zhou J, et al. Review and consensus recommendations on clinical APT-weighted imaging approaches at 3T: Application to brain tumors. Magn Reson Med. 2022;88(2):546–74. 35452155 10.1002/mrm.29241 PMC9321891

[25] Welker K, et al. ASFNR recommendations for clinical performance of MR dynamic susceptibility contrast perfusion imaging of the brain. AJNR Am J Neuroradiol. 2015;36(6):E41–51. 25907520 10.3174/ajnr.A4341 PMC5074767

[26] Cho NS, et al. A multi-reader comparison of normal-appearing white matter normalization techniques for perfusion and diffusion MRI in brain tumors. Neuroradiology. 2023;65(3):559–68. 36301349 10.1007/s00234-022-03072-y PMC9905164

[27] Zhang Z, et al. Amide proton transfer-weighted magnetic resonance imaging of human brain aging at 3 Tesla. Quant Imaging Med Surg. 2020;10(3):727–42. 32269932 10.21037/qims.2020.02.22 PMC7136735

[28] Oh JH, et al. Added Value of Chemical Exchange-Dependent Saturation Transfer MRI for the Diagnosis of Dementia. Korean J Radiol. 2021;22(5):770–81. 33543845 10.3348/kjr.2020.0700 PMC8076822

[29] Fujita S, et al. Characterization of Brain Volume Changes in Aging Individuals With Normal Cognition Using Serial Magnetic Resonance Imaging. JAMA Netw Open. 2023;6(6):e2318153. 37378985 10.1001/jamanetworkopen.2023.18153 PMC10308250

[30] Paech D, et al. Relaxation-compensated amide proton transfer (APT) MRI signal intensity is associated with survival and progression in high-grade glioma patients. Eur Radiol. 2019;29(9):4957–67. 30809720 10.1007/s00330-019-06066-2

[31] Regnery S, et al. Chemical exchange saturation transfer MRI serves as predictor of early progression in glioblastoma patients. Oncotarget. 2018;9(47):28772–83. 29983895 10.18632/oncotarget.25594 PMC6033360

[32] Joo B, et al. Amide proton transfer imaging might predict survival and IDH mutation status in high-grade glioma. Eur Radiol. 2019;29(12):6643–52. 31175415 10.1007/s00330-019-06203-x PMC6859837

[33] Kroh F, et al. Semi-solid MT and APTw CEST-MRI predict clinical outcome of patients with glioma early after radiotherapy. Magn Reson Med. 2023. 10.1002/mrm.29746 37317562

[34] Liu J, et al. Diagnostic performance of multiparametric MRI in the evaluation of treatment response in glioma patients at 3T. J Magn Reson Imaging. 2020;51(4):1154–61. 31430008 10.1002/jmri.26900

[35] Park KJ, et al. Added value of amide proton transfer imaging to conventional and perfusion MR imaging for evaluating the treatment response of newly diagnosed glioblastoma. Eur Radiol. 2016;26(12):4390–403. 26883333 10.1007/s00330-016-4261-2

[36] Park YW, et al. Differentiation of recurrent diffuse glioma from treatment-induced change using amide proton transfer imaging: incremental value to diffusion and perfusion parameters. Neuroradiology. 2021;63(3):363–72. 32879995 10.1007/s00234-020-02542-5

[37] Zhou J, et al. Amide proton transfer (APT) contrast for imaging of brain tumors. Magn Reson Med. 2003;50(6):1120–6. 14648559 10.1002/mrm.10651

[38] Paech D, et al. Assessing the predictability of IDH mutation and MGMT methylation status in glioma patients using relaxation-compensated multipool CEST MRI at 7.0 T. Neuro Oncol. 2018;20(12):1661–71. 29733378 10.1093/neuonc/noy073 PMC6231210

[39] Goerke S, et al. Clinical routine acquisition protocol for 3D relaxation-compensated APT and rNOE CEST-MRI of the human brain at 3T. Magn Reson Med. 2021;86(1):393–404. 33586217 10.1002/mrm.28699

