# Supplementary material for: Assessment of multimodal CEST, perfusion and diffusion MRI for predicting clinical outcome of patients with diffuse glioma following surgery at baseline before radiotherapy
Source: Cancer Imaging. 2026 Mar 28;26:61. doi: 10.1186/s40644-026-01022-y (PMC13151286; doi:10.1186/s40644-026-01022-y)
Supplement: Supplementary file 1 — Supplementary Material 1 [file 40644_2026_1022_MOESM1_ESM.docx]

**Supplementary Table**

| **Characteristic** | |  |  | **Number (n)** | | **Percentage** |
| --- | --- | --- | --- | --- | --- | --- |
|  |  |  |  |  |  |  |
| Median age at diagnosis | | Median 62.0 [95% CI: 58.5; 64.0] | | 78 |  | 100% |
| Sex |  | Female / male | | 27 / 51 |  | 34.6% / 65.4% |
|  |  |  |  |  |  |  |
| RANO response after completion of RT | | Stable disease (SD)* | | 58 |  | 74.4% |
|  |  | Pseudo progression (PP) | | 12 |  | 15.4% |
|  |  | Progressive disease (PD) | | 15 |  | 19.2% |
|  |  | N/A |  | 5 |  | 6.4% |
|  |  |  |  |  |  |  |
| Status |  | Alive |  | 29 |  | 37.2% |
|  |  | Lost to FU |  | 3 |  | 3.8% |
|  |  | PFS reached |  | 61 |  | 78.2% |
|  |  | OS reached |  | 46 |  | 59.0% |
|  |  |  |  |  |  |  |
| Survival |  | Median FU |  | 23.2 months (min. 0.6; max. 30.4) | | |
|  |  | Median PFS |  | 6.04 months (95%-CI: 4.60 - 10.45) | | |
|  |  | Median OS |  | 11.58 months (95%-CI: 8.41; 14.36) | | |
|  |  |  |  |  |  |  |
| KPS |  | <70% |  | 9 |  | 11.5% |
|  |  | ≥70% |  | 69 |  | 88.5% |
|  |  |  |  |  |  |  |
| Treatment for | | Initial disease |  | 65 |  | 83.3% |
|  |  | Progressive disease | | 13 |  | 16.7% |
|  |  |  |  |  |  |  |
| Therapy |  | Radiation |  | 22 |  | 28.2% |
|  |  | Chemoradiation | | 56 |  | 71.8% |
|  |  | Debulking surgery | | 50 |  | 64.1% |
|  |  |  |  |  |  |  |
| Diagnosis |  | GBM |  | 51 |  | 65.4% |
|  |  | Midline-GBM |  | 3 |  | 3.8% |
|  |  | Gliosarcoma |  | 7 |  | 9.0% |
|  |  | Astrocytoma |  | 9 |  | 11.5% |
|  |  | Oligodendroglioma | | 7 |  | 9.0% |
|  |  | Pleomorphic Xanthoastrocytoma | | 1 |  | 1.3% |
|  |  |  |  |  |  |  |
| WHO |  | II |  | 10 |  | 12.8% |
|  |  | III |  | 5 |  | 6.4% |
|  |  | IV |  | 63 |  | 80.8% |
|  |  |  |  |  |  |  |
| IDH status |  | IDHwt |  | 59 |  | 75.6% |
|  |  | IDHmut |  | 16 |  | 20.5% |
|  |  | n/a |  | 3 |  | 3.8% |
|  |  |  |  |  |  |  |
| MGMT promotor methylation | | Yes |  | 38 |  | 48.7% |
|  |  | No |  | 28 |  | 35.9% |
|  |  | n/a |  | 12 |  | 15.4% |
|  |  |  |  |  |  |  |

**Table S1:** Characteristics of study participants. Listed are: Age at diagnosis for females and males, type of therapy response according to the response assessment in neuro-oncology (RANO) criteria after completion of radiotherapy (RT), viability status at the end of data acquisition, karnovsky performance score (KPS) of ≥ 70 or < 70, treatment for initial or relapsing disease, therapy type following the baseline scan, Glioma type according to the WHO classification of primary CNS neoplasms from 2016, WHO glioma grade, IDH mutation status and presence of MGMT promotor methylation. FU = Follow-up; PFS = Progression-free survival; OS = Overall survival; WHO = World health organization (WHO); IDH = Isocitrate dehydrogenase isotypes 1 and 2; wt = Wild type; mut = Mutated; MGMT = O-6-methylguanine-DNA methyltransferase; n/a = Not available. *SD = Stable disease, includes all patients with Pseudoprogression.

**Supplementary Figure**


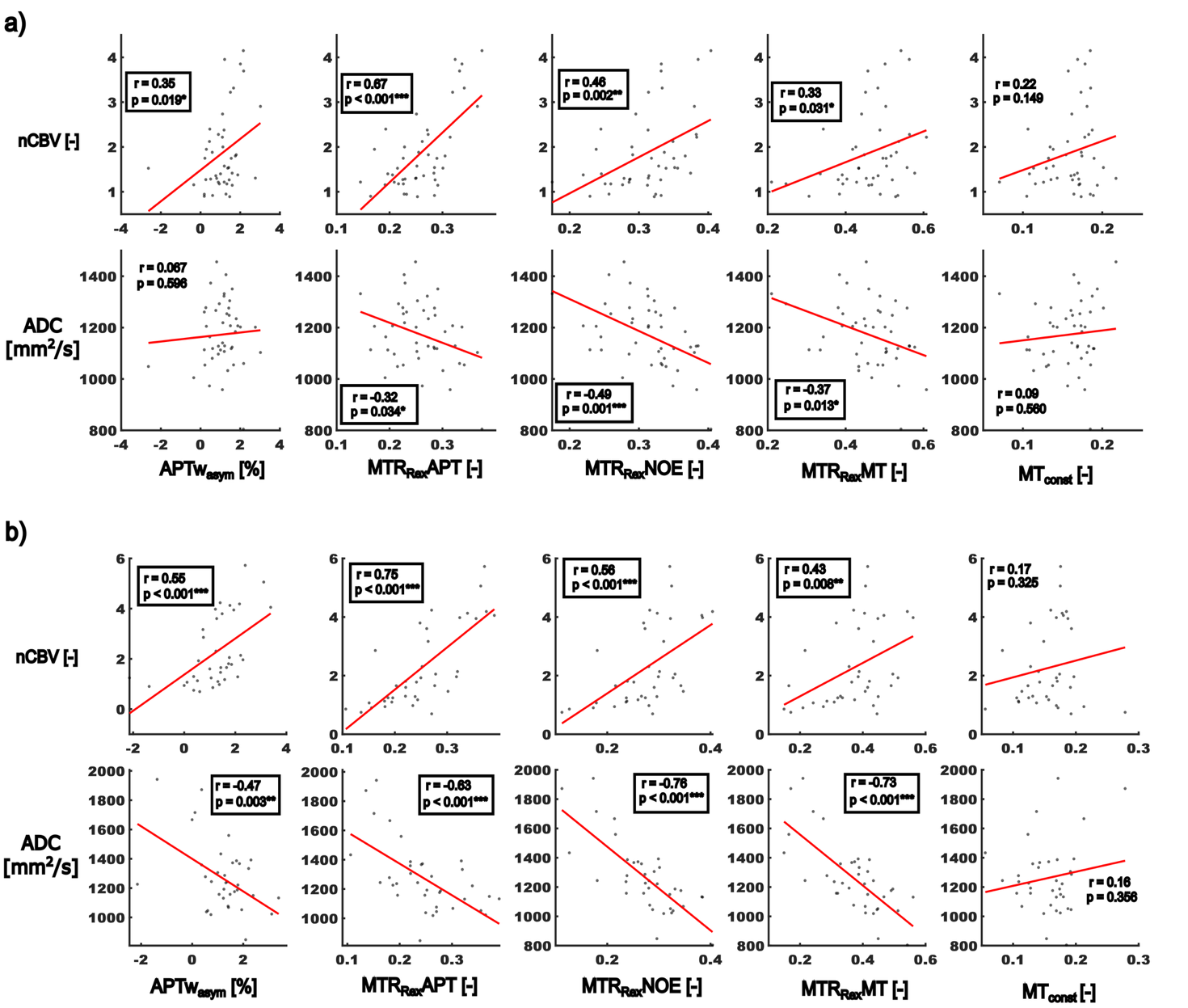


**Figure S1:** Pearson correlation of normalized cerebral blood volume (nCBV) and apparent diffusion coefficient (ADC) maps with CEST-contrasts. Assessments were performed in study participants with an initial diagnosis of glioma and age ≤ 70 years at baseline before radiotherapy (sub-cohort). The data of five participants without perfusion imaging was censored in this analysis. a) Results for whole tumor volumes (WT) assessed in 44 participants. b) Results for contrast-enhancing (CE) tumor volumes assessed in 37 participants with residual contrast enhancement on MRI. Given are the correlation coefficient r with respective p-values. [-] = Arbitrary units. P ≤ 0.05, ≤ 0.01 and ≤ 0.001 are indicated with “*”, “**” and “***” respectively.
